# The renal baroreflex: A systematic review and meta‐analysis in healthy and hypertensive animals

**DOI:** 10.14814/phy2.70547

**Published:** 2025-09-10

**Authors:** Maaike van Ochten, Wisal El Fathi, Esmee M. E. Bovee, Marc E. A. Spaanderman, Carlijn R. Hooijmans, Joris van Drongelen

**Affiliations:** ^1^ Department of Obstetrics and Gynecology Radboud University Medical Center Nijmegen The Netherlands; ^2^ Department of Gynecology and Obstetrics Maastricht University Medical Center Maastricht The Netherlands; ^3^ Department of Anesthesiology, Pain and Palliative Medicine Radboud University Medical Center Nijmegen The Netherlands

**Keywords:** hypertension, renal baroreflex, renin, renin‐angiotensin‐aldosterone system

## Abstract

The renal baroreflex describes the dose‐dependent relation between renal pressure and renin release. Former studies have approximated this relation through animal experiments, but the exact shape of the response curve and its alteration by hypertension remain unclear. Therefore, we conducted a systematic review and meta‐analysis on the renal baroreflex in healthy and hypertensive animals. Studies retrieved from Pubmed and Embase were screened according to predefined criteria. Data regarding study design and outcome data were extracted, and risk of bias was assessed. Dose–response meta‐analysis was conducted on the relationship between renal blood pressure and renin release. Subgroup analyses were conducted for species, sex, outcome units, and experimental setup. From 1508 studies, 55 were included in the systematic review, and 30 in the meta‐analysis. Risk of bias was high in most studies. Our meta‐analysis shows that in healthy animals, renin decreases linearly with increasing renal blood pressure, reaching a plateau above a threshold pressure of 93 ± 2 mmHg. In hypertensive animals, the linear slope is diminished, the threshold is comparable, and the plateau level is slightly decreased as compared to healthy animals. Despite some limitations, our findings suggest that high blood pressure in hypertensive animals blunts the renal baroreflex by resetting and suppression of the renin response to changes in blood pressure.

## 1 INTRODUCTION

The renin‐angiotensin‐aldosterone system (RAAS) is a key modulator in volume homeostasis and arterial blood pressure control. Renin, the first hormone in the cascade of the RAAS, is released by the juxtaglomerular cells orchestrated by four major pathways: activation of the sympathetic nervous system, reduced levels of volume regulatory hormones (such as atrial natriuretic peptide and angiotensin II), reduced pre‐urinary salt passage at the site of the macula densa, and low renal perfusion pressure (Peti‐Peterdi & Harris, 2010; Wagner & Kurtz, 1998). Release of renin as a response to a decrease in renal pressure is called the renal baroreflex.

**FIGURE 1:**
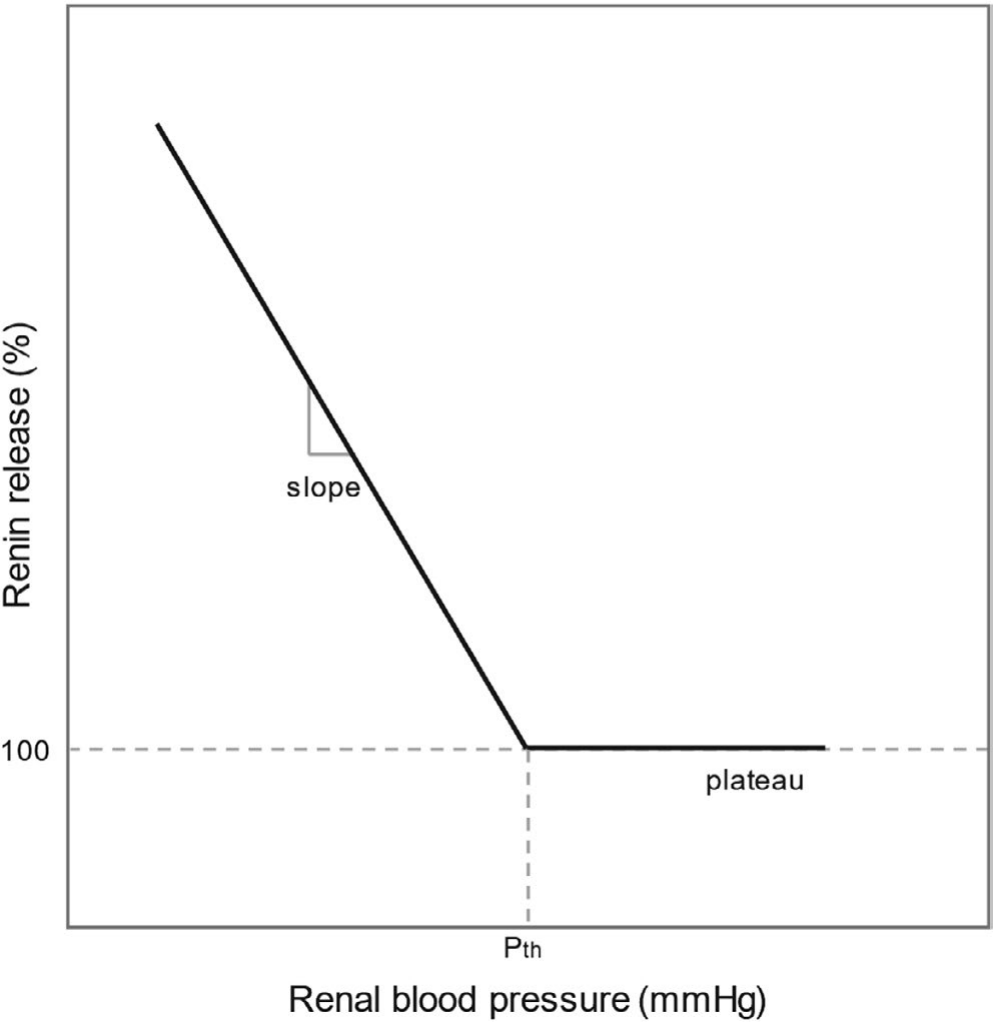
An example of the shape of the renin‐stimulus response curve, which describes the influence of renal arterial pressure on renin release. Pth, threshold pressure.

The dose‐dependent relation between renal pressure and renin release by the renal baroreflex can be described by a renin stimulus–response curve (RSRC). The shape of this curve is determined by three main characteristics: (1) the threshold pressure below which renin release is pressure‐dependent; (2) the slope of the linear relation between renin release and renal blood pressure below the threshold pressure; and (3) the plateau level of renin above the threshold pressure (Figure 1). Different studies have approximated the RSRC through animal experiments, but there is neither agreement on the values of the three main characteristics nor to what extent they are dependent on other variables, such as animal species, salt diet, or chronically high blood pressure (Kirchheim et al., 1989). Profound understanding and accurate quantification of the renin response to stimuli is essential to better predict and anticipate on conditions that affect blood pressure, especially in individuals with endangered kidney function. The aim of this study is to approximate the dose‐dependent relation between renal pressure and renin release in normotensive and hypertensive animals. To this end, we performed a systematic review and meta‐regression analysis.

## 2 METHODS

We performed a systematic review and meta‐analysis to define the dose–response relation between renal blood pressure and renin release. This research follows the PRISMA guidelines and was registered in PROSPERO (registration ID: CRD42023444676) (Booth et al., 2012; Moher et al., 2015).

### 2.1 Search strategy

We conducted the systematic search in PubMed and Embase to collect studies on the relation between renal blood pressure and renin release. These electronic databases were used to find studies published up to and until November 27, 2024. We used the following components for the search: (1) “kidney”/“renal circulation”, (2) “renin”, (3) “blood pressure”, and (4) “baroreceptor”. The complete search strategy is shown in Appendix A. The search strategy was constructed with the advice of a librarian (OYC) from the Radboud University.

### 2.2 Study selection

Studies were screened for eligibility by two independent reviewers (MO and WEF). Any disagreements were solved by mutual consensus. Screening was performed in Covidence (n.d.) and consisted of two phases. First, studies were assessed based only on their title and abstract. Second, the full texts of potentially eligible studies were screened. Studies were included if the study objects were (non‐human) animals, only renal blood pressure was altered, renin concentration was measured at a minimum of two different renal blood pressure levels, and if renin was measured within 30 min of the blood pressure adjustment. Studies were excluded if blood pressure was altered by drugs or if there was no adequate control or baseline measurement. We did not exclude studies based on language.

### 2.3 Data extraction

Two reviewers (MO and EB) extracted the study characteristics and experimental outcomes from the selected studies in duplicate. Discrepancies were solved by mutual consensus. The primary outcome to extract was renin release at a certain blood pressure level. Secondary outcomes consisted of information on the RSRC (threshold, slope, and plateau). Data on the dose–response relation between renal blood pressure and renin were documented as mean blood pressure level, mean renin level, and corresponding standard error (SE). Two studies reported data on individual animals (Imbs et al., 1970; Kirchheim et al., 1987). For these studies, we calculated the corresponding mean and SE. One study reported its outcomes as the logarithm of the outcome (Bock et al., 1992): these values were converted to the actual values. If multiple renin outcomes were reported per experimental group, the most common outcome reported within other studies was used in the main analysis. Data that were only presented graphically were extracted by the use of online software (Rohatgi, 2022). When outcome measure data were missing or not clearly readable, we attempted to contact authors for additional information (up to two emails). If the data could not be obtained, we used a conservative estimate when possible. Furthermore, we extracted data on study design (number of animals and type of study), animal characteristics (species, strain, sex, age, weight, and baseline renal blood pressure), experimental setup (diet, in vivo or isolated, sampling method), and outcome measures (reported units). Renin values reported as renin activity were categorized as plasma renin activity (PRA); values reported as renin secretion rate (RSR) or renin secretion were categorized as renin release (RR).

### 2.4 Data synthesis

#### 2.4.1 Dose–response meta‐analysis in healthy animals

Dose–response meta‐analysis was conducted to determine the relation between renal blood pressure and renin release. The statistical analyses were performed with the statistical software R (version 4.1.3) (R Core Team, 2022). We used the dosresmeta package (Crippa & Orsini, 2016) to conduct a two‐stage dose–response meta‐analysis using a restricted cubic spline (RCS) model. An RCS model is a statistical tool to characterize a nonlinear dose–response relation between a continuous exposure and an outcome by fitting piecewise polynomial functions with constraints at specified knots. This allows us to compare observed dose–response relations in a relative manner. In this study, we used an RCS model with three knots, located at the 10th, 50th, and 90th percentiles of the overall blood pressure range (Harrel, 2015). To adequately define the spline segments at these knots, only studies with at least four data points with measures of variation could be included in the meta‐analysis. The resulting function was plotted compared to a reference blood pressure level where the relative renin level is set to be zero. This reference blood pressure was chosen equal to the mean baseline renal arterial pressure (MRAP) of the comparisons included in the meta‐analysis. Renin levels were expressed as standardized mean difference (SMD) and the corresponding 95 % confidence interval (CI). Subsequently, SMD values were converted to percental values, where the renin values of healthy animals at the baseline MRAP of 109 mmHg are set to be 100%. Only studies that measured renin at the expected plateau level (MRAP >105 mmHg) were included in this conversion (*N* = 30).

#### 2.4.2 Dose–response meta‐analysis in hypertensive animals

The meta‐analysis in hypertensive animals was conducted with a similar approach as the meta‐analysis in healthy animals. To improve interpretation of the differences between the hypertensive and healthy groups, the SMD was converted to a percentage. In this analysis, we only included studies that evaluated the dose–response relation in both hypertensive and healthy animals (*N* = 5). Renin was normalized to the healthy animals in those studies, where renin values of healthy control animals at their baseline MRAP of 110 mmHg were set to be 100%.

#### 2.4.3 Meta‐analysis on threshold pressure and plateau levels

In addition to the dose–response meta‐analysis, we performed post hoc meta‐analyses regarding the difference in threshold pressure and renin plateau levels between healthy and hypertensive animals. These variables were determined based on the results of our dose–response meta‐analysis for healthy and hypertensive animals separately. In addition, we determined the threshold pressure and renin plateau levels based on the individual dose response curve in studies with a direct comparison of healthy and hypertensive animals.

A linear regression was performed for the low‐pressure range of renal artery pressure (below 90 mmHg). In the high‐pressure range (above 105 mmHg), all renin values were averaged to define the plateau level. The threshold pressure was identified as the point where the regression line for the low‐pressure range intersects with the plateau (Imagawa et al., 1984; Kirchheim et al., 1985, 1987; Porter, 1990; Wende et al., 1993). A random effects model was used to determine the overall ratio of means (ROM) between healthy and hypertensive comparisons, using the meta package in R (Balduzzi et al., 2019). The ROM was only determined for studies that measured both healthy and hypertensive animals (*N* = 5).

### 2.5 Subgroup and sensitivity analyses

Predefined subgroup analyses (see protocol in PROSPERO: CRD42023444676) were conducted for animal species, sex, and diet in case of a minimum of 10 comparison studies per subgroup. In addition, subgroup analyses based on reported renin outcomes and experimental setup (in vivo or isolated) were conducted, as variation in these factors was observed across studies and was considered to potentially influence the results. The knots and reference blood pressure level were adjusted for each subgroup, dependent on the blood pressure range and baseline renal blood pressure (see Appendix D, Table D1). Sensitivity analyses were performed to evaluate the effect of publication date, risk of bias, and type of study on the dose–response relation. Furthermore, we compared an RCS model with four knots located at the 5th, 35th, 65th, and 95th percentiles of the overall blood pressure range (Harrel, 2015) to the RCS with three knots. We compared the fit of the two RCS models by evaluating the Akaike Information Criterion (AIC) and Bayesian Information Criterion (BIC) values, selecting the model with the lower AIC and BIC as the better‐fitting model (Shmueli, 2010). Heterogeneity between the studies was evaluated using the *I* ^2^ statistic.

### 2.6 Risk of bias

Risk of bias was assessed by two independent reviewers (MO and WEF). We used two different tools to evaluate the risk of bias. The ROBINS‐I tool (Sterne et al., 2016) was used to assess studies that only included an experimental group, where the animals are their own control subjects. The SYRCLE RoB tool (Hooijmans et al., 2014) was used to assess studies that used an experimental group with an independent control group. Both tools were adjusted to make them suitable for the study designs included in our analysis.

#### 2.6.1 ROBINS‐I tool

We adjusted the ROBINS‐I tool based on the suggestions from table 25.5a in the Cochrane handbook (Sterne et al., 2023) for baseline control studies. The following adjustments were made:

Questions under the domain “bias due to confounding” were altered to: “1.1 Is there potential for confounding of the effect of intervention in this study due to the carry‐over effect?”; “1.2 Were the measurements of outcome made at sufficient pre‐intervention time points to permit characterization of pre‐intervention trend and patterns?”; “1.3 Were there extraneous events or changes in context around the time of intervention that could have influenced the outcome?”; “1.4 Did the study authors use an appropriate analysis method that accounts for time trend and patterns, and controls for all the important confounding domains?”.

Elements of ROBINS‐I deemed not relevant for our research question and study designs were excluded, for example, the domain “bias in selection of participants into the study”, and questions 4.3–4.6 (bias due to deviations from intended interventions) were removed.

Question 4.1 was rephrased into “Was there appropriate accounted for the effects of any preparatory (pre‐interruption) phases of the intervention?”.

Two additional question were added: “5.6 Was outcome data missing for whole groups of animals as well as individual animals?” and “6.4 Were the methods of outcome assessment comparable before and after the intervention?”.

Signaling questions of the ROBINS‐I can be scored with (1) Yes; (2) Probably yes; (3) Probably no; (4) No; and (5) No information. In the tool, a response marked in green indicates low risk of bias, responses in red suggest high risk of bias, and “No information” indicates unknown risk of bias. The ROBINS‐I tool can be found in Appendix B.1.

#### 2.6.2 SYRCLE tool

Signaling questions of the SYRCLE tool were scored with “yes”, indicating a low risk of bias, “no”, indicating a high risk of bias or “unclear”, indicating an unknown risk of bias. In the domain assessing the risk of other biases (domain 6), the risk of a potential carry over effect was assessed. Furthermore, three questions related to reporting quality were added to the tool: “1. Did the authors apply randomization at any level?”; “2. Was there any level of blinding?” and “3. Was there a sample size calculation reported?”. The adjusted SYRCLE tool can be found in Appendix B.2.

## 3 RESULTS

### 3.1 Study and data selection

**FIGURE 2:**
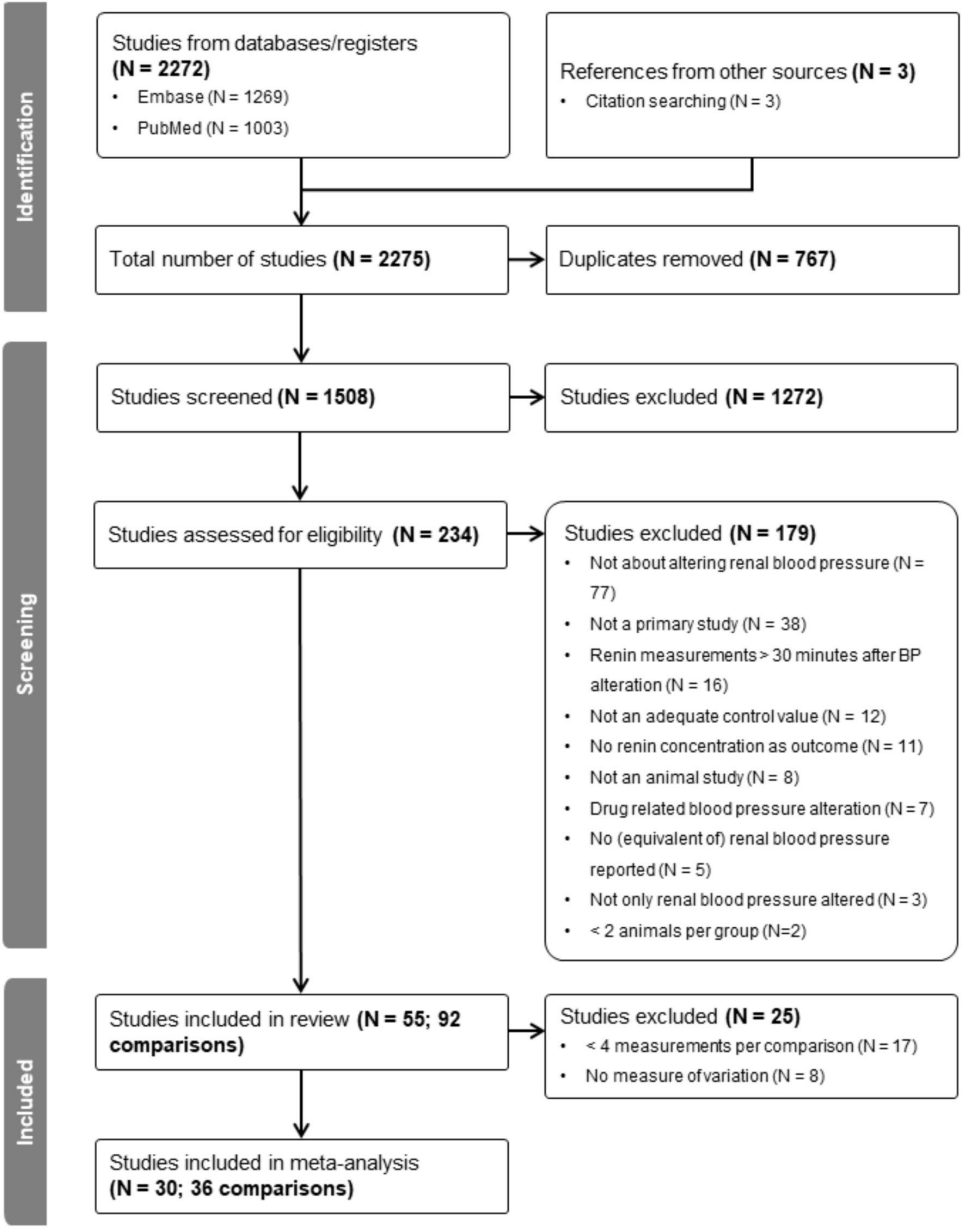
PRISMA‐P flowchart.

Our comprehensive search yielded 2275 references. After deduplication, 1508 references were screened, and 55 references could be included in our systematic review (Figure 2). Studies were excluded if they were not about altering renal blood pressure (*N* = 77), were not primary studies (*N* = 38), measured renin more than 30 min after the blood pressure alteration (*N* = 16), did not report an adequate control or baseline measurement (*N* = 12), did not report renin as an outcome (*N* = 11), were not animal studies (*N* = 8), altered blood pressure by drugs (N = 7), did not report renal blood pressure (*N* = 5), did not only alter renal blood pressure (*N* = 3), or did not include more than one animal in their experimental groups (*N* = 2).

In the meta‐analysis, we excluded studies that had less than four measurements per comparison (*N* = 17) or did not report a measure of variation like the SD or SE (*N* = 8). Studies with less than four measurements often did not measure renin at higher blood pressure levels, or only measured renin at two different blood pressure levels. As a result, the meta‐analysis included 30 studies (Berthold et al., 1999; Bertolino et al., 1994, 1996; Braun et al., 1996, 1998; Ehmke et al., 1992, 1993; Fan et al., 1994; Finke et al., 1983; Freeman et al., 1982; Imagawa et al., 1984; Imbs et al., 1970; Kirchheim et al., 1985, 1987; Knoblich et al., 1996; Kopecky et al., 2001; Kurt et al., 2011; Medeiros et al., 1992; Persson et al., 1993; Porter, 1990; Salomonsson et al., 1992; Scholz, Gotz, et al., 1994; Scholz, Hamann, et al., 1994; Scholz & Kurtz, 1992; Schweda et al., 2005; Seeliger et al., 1999; Wagner et al., 2010; Wende et al., 1993; Wideman Jr et al., 1993).

### 3.2 Study characteristics

#### 3.2.1 Healthy animals

An overview of the characteristics of the 55 studies containing 92 comparisons in healthy animals can be found in Table 1. Most experimental comparisons were made in rats (*N* = 41) and dogs (*N* = 30). Experiments were either performed in vivo (*N* = 62) or in isolated kidneys or glomeruli (N = 30). Most comparisons used male animals (*N* = 37); only a few comparisons used female animals (*N* = 10). Other comparisons used both sexes (*N* = 15) or did not specify the sex of the experimental animals (*N* = 30). Studies reported their renin measurements either as plasma renin activity (PRA) (*N* = 47), plasma renin concentration (PRC) (*N* = 6), or an equivalent of renin release (RR) (*N* = 39). A visual overview of the study characteristics can be found in Appendix C. The baseline MAP of the healthy animals was 112 ± 18 mmHg. Some studies (*N* = 9) determined an RSRC with a threshold pressure (Bertolino et al., 1994; Ehmke et al., 1992; Farhi et al., 1982, 1987; Imagawa et al., 1984; Kirchheim et al., 1987; Porter, 1990, 1992; Wende et al., 1993; Wideman Jr et al., 1993). The mean threshold pressure in these studies was 91 ± 8 mmHg.

**TABLE 1:** Study characteristics of healthy comparisons.

Study	Species	Strain	Sex	Age	Weight	Baseline BP (mmHg)	Diet	Setup	Outcome	Sampling method	N	MA
Beierwaltes et al. (1995)	Rat	Sprague–Dawley	Male	n.s.	250–350 g	RPP: 104 ± 3	Fasting	In vivo	PRC	Venous	12	No
	Rat	Sprague–Dawley	Male	n.s.	250–350 g	RPP: 104 ± 3	Fasting	In vivo	RSR	Venous	12	No
Beierwaltes et al. (2002)	Mouse	Wildtype (C57BL/6J)	n.s.	n.s.	n.s.	basal RP: 86 ± 1	n.s.	In vivo	PRC	Venous	6	No
Berl et al. (1979)	Dog	Mongrel	n.s.	n.s.	20–30 kg	RPP: 143 ± 8	Fasting	In vivo	PRA	n.s.	6	No
Berthold et al. (1999)	Dog	Foxhound	Both	n.s.	28–31 kg	MAP: 92 ± 5	100 mEq	In vivo	RR	Venous–Arterial difference	5	Yes
Bertolino et al. (1994)	Rat	Lyon	Male	13 weeks	268 ± 4 g	MRAP: 114 ± 2	Standard	In vivo	PRC	Arterial	10	Yes
Bertolino et al. (1996)	Rat	Lyon	Male	14–16 weeks	n.s.	MRAP: 111 ± 1	Standard	In vivo	PRC	Arterial	11	Yes
Binder et al. (1992)	Lamb	n.s.	n.s.	136 + −1 days GA	n.s.	MAP LB: 48 ± 1	n.s.	In vivo	PRA	Arterial	11	No
	Lamb	n.s.	n.s.	141 + −1 days GA (1–7 days after delivery)	n.s.	MAP LB: 64 ± 2	Standard	In vivo	PRA	Arterial	3	No
Bock et al. (1992)	Rabbit	New Zealand White	n.s.	n.s.	1–1.5 kg	n.s.	n.s.	Isolated	log(RSR)	Arteriolar	6	No
	Rabbit	New Zealand White	n.s.	n.s.	1–1.5 kg	n.s.	n.s.	Isolated	log(RSR)	Arteriolar	5	No
Braun et al. (1996)	Rat	n.s.	Male	3 months	417.2 ± 9.4 g	RPP: 112.1 ± 2.8	Standard	In vivo	PRA	n.s.	6	Yes
	Rat	n.s.	Male	9 months	490.9 ± 7.9 g	RPP: 111.5 ± 3.6	Standard	In vivo	PRA	n.s.	7	Yes
Braun et al. (1998)	Rat	Wistar‐Kyoto	Male	n.s.	300–400 g	MAP: 108 ± 2	Standard	In vivo	PRA	n.s.	6	Yes
Data et al. (1978)	Dog	Mongrel	n.s.	n.s.	16.5–30 kg	FAP: 121.1 ± 4.0	n.s.	In vivo	PRA	Venous	n.s.	No
	Dog	Mongrel	n.s.	n.s.	16.5–30 kg	FAP: 124.9 ± 6.8	n.s.	In vivo	PRA	Venous	n.s.	No
Ehmke et al. (1992)	Dog	Foxhound	Both	Adult	19–28 kg	MRAP: 108 ± 4	100 mEq	In vivo	RR	Venous–Arterial difference	8	No
	Dog	Foxhound	Both	Adult	19–28 kg	MRAP: 108 ± 4	100 mEq	In vivo	RR	Venous–Arterial difference	4	Yes
Ehmke et al. (1993)	Dog	Foxhound	Both	Adult	19–24 kg	RPP: 101 ± 4	100 mEq	In vivo	PRA	Arterial	6	Yes
Fan et al. (1994)	Sheep	Mixed‐breed	Female	n.s.	n.s.	RPP: 83 ± 2	100 mEq	In vivo	PRA	Venous	5	Yes
	Sheep	Mixed‐breed	Female	n.s.	n.s.	RPP: 83 ± 2	100 mEq	In vivo	RSR	Venous–Arterial difference	5	Yes (sub)
Farhi et al. (1982)	Dog	Mongrel	Male	n.s.	24–34 kg	n.s.	Low salt diet	In vivo	PRA	Arterial	5	No
Farhi et al. (1983)	Dog	Mongrel	Male	n.s.	25–35 kg	n.s.	80 mEq	In vivo	PRA	n.s.	5	No
Farhi et al. (1987)	Dog	Mongrel	Male	n.s.	25–35 kg	n.s.	80 mEq	In vivo	Normalized PRA	n.s.	4	No
	Dog	Mongrel	Male	n.s.	25–35 kg	n.s.	Low salt diet	In vivo	Normalized PRA	n.s.	5	No
Finke et al. (1983)	Dog	Foxhound	n.s.	Adult	23.8 ± 0.6 kg	MRAP: 106 ± 3.9	4 mmol/kg/day	In vivo	PRA	Arterial	6	Yes
	Dog	Foxhound	n.s.	Adult	23.8 ± 0.6 kg	MRAP: 106 ± 3.9	4 mmol/kg/day	In vivo	RR	Venous–Arterial difference	6	Yes (sub)
Freeman et al. (1982)	Dog	Mongrel	Female	Adult	n.s.	RPP: 109 ± 5	60 mEq	In vivo	PRA	Arterial	5	Yes
Gerl et al. (2013)	Mouse	n.s.	Both	12–16 weeks	n.s.	n.s.	n.s.	Isolated	PRC	Venous	5	No
Gerl et al. (2015)	Mouse	n.s.	Both	12–24 weeks	n.s.	n.s.	Standard	Isolated	PRC	Venous	5	No
Imagawa et al. (1984)	Rat	Wistar	Male	n.s.	290–330 g	RAP: 137.4 ± 3.4	Standard	In vivo	PRA	Arterial	12	Yes
Imbs et al. (1970)	Dog	n.s.	Both	n.s.	13–27 kg	n.s.	Fasting	In vivo	PRA	Venous	10	Yes
	Dog	n.s.	Both	n.s.	13–27 kg	n.s.	Fasting	In vivo	Renin secretion	Venous	6	Yes (sub)
Jensen et al. (1998)	Rat	Sprague–Dawley	Male	n.s.	250–300 g	n.s.	Standard	Isolated	RSR	Venous–Arterial difference	5	No
Johns (1985)	Cat	n.s.	Male	n.s.	3.1–5.1 kg	RPP: 146 ± 4	n.s.	In vivo	RSR	Venous–Arterial difference	5	No
Jones‐Dombi et al. (1993)	Rat	Sprague–Dawley	Male	Adult	220–320 g	n.s.	Standard	Isolated	RSR	n.s.	16	No
	Rat	Sprague–Dawley	Male	Adult	220–320 g	n.s.	Standard	Isolated	RSR	n.s.	14	No
	Rat	Sprague–Dawley	Male	Adult	220–320 g	n.s.	Standard	Isolated	RSR	n.s.	14	No
	Rat	Sprague–Dawley	Male	Adult	220–320 g	n.s.	Standard	Isolated	RSR	n.s.	14	No
	Rat	Sprague–Dawley	Male	Adult	220–320 g	n.s.	Standard	Isolated	RSR	n.s.	10	No
	Rat	Sprague–Dawley	Male	Adult	220–320 g	n.s.	Standard	Isolated	RSR	n.s.	9	No
	Rat	Sprague–Dawley	Male	Adult	220–320 g	n.s.	Standard	Isolated	RSR	n.s.	10	No
	Rat	Sprague–Dawley	Male	Adult	220–320 g	n.s.	Standard	Isolated	RSR	n.s.	4	No
Kaloyanides et al. (1973)	Dog	Mongrel	n.s.	n.s.	15–25 kg	RAP: 99 ± 4	Fasting	Isolated	Renin activity	Venous–Arterial difference	11	No
Kirchheim et al. (1985)	Dog	Foxhound	n.s.	Adult	21.6 ± 1.5 kg	MRAP: 113.9 ± 1.0	100 mEq	In vivo	PRA	Arterial	7	Yes
Kirchheim et al. (1987)	Dog	Foxhound	n.s.	Adult	21.8 ± 0.52 kg	n.s.	5 mmol/kg/day	In vivo	PRA	Venous	1	Yes
Knoblich et al. (1996)	Rat	Sprague–Dawley	Male	n.s.	275‐400 g	RPP: 121.1	Fasting	In vivo	PRA	Arterial	18	Yes
Kopecky et al. (2001)	Rat	Nondiabetic BB/OK	Male	225 + −11 days	396 ± 16 g	RPP: 109 ± 4	Standard	In vivo	PRA	n.s.	5	Yes
	Rat	Wistar	Male	225 days	653 ± 26 g	RPP: 115 ± 4	Standard	In vivo	PRA	n.s.	9	Yes
Kurt et al. (2011)	Mouse	Cx45	Male	n.s.	n.s.	n.s.	n.s.	Isolated	RSR	Venous–Arterial difference	4	Yes
Machura et al. (2013)	Mouse	Wildtype	Male	8–12 weeks	n.s.	n.s.	Standard	Isolated	RSR	Venous	3	No
Medeiros et al. (1992)	Rat	Lyon	Male	7 weeks	138 ± 7 g	n.s.	Standard	Isolated	RR	Venous	6	Yes
Nafz et al. (1997)	Dog	Foxhound	Both	Adult	18.5–15 kg	RAP: 109 ± 5	Fasting	In vivo	PRA	Venous	8	No
Nobiling et al. (1990)	Rat	Sprague–Dawley	Male	n.s.	160–180 g	n.s.	n.s.	Isolated	RR	Venous	n.s.	Yes
Osborn et al. (1982)	Dog	Mongrel	n.s.	n.s.	16–23 kg	RPP: 137 ± 5	n.s.	In vivo	RSR	Venous–Arterial difference	10	No
	Dog	Mongrel	n.s.	n.s.	16–23 kg	RPP: 140 ± 10	n.s.	In vivo	RSR	Venous–Arterial difference	4	No
Osborn et al. (1984)	Dog	Mongrel	Both	n.s.	15–25 kg	FAP: 135	Fasting	In vivo	RSR	Venous–Arterial difference	)	No
Persson et al. (1993)	Dog	Foxhound	Female	n.s.	22 ± 1 kg	RAP: 100	Standard	In vivo	RR	Venous–Arterial difference	6	Yes
Peters et al. (1994)	Dog	Beagle	Female	n.s.	10–16 kg	RPP: 129 ± 7	5 mmol/kg/day	In vivo	PRA	Arterial	7	No
Porter (1990)	Rat	Wistar‐Kyoto	n.s.	6–9 weeks	120–175 g	RPP: 113	n.s.	In vivo	PRA	Arterial	5	No
	Rat	Wistar‐Kyoto	n.s.	6–9 weeks	120–175 g	RPP: 107	n.s.	In vivo	PRA	Arterial	5	No
	Rat	Wistar‐Kyoto	n.s.	6–9 weeks	120–175 g	RPP: 112.3	n.s.	In vivo	PRA	Arterial	12	No
	Rat	Wistar‐Kyoto	n.s.	14–16 weeks	210–320 g	RPP: 110 ± 4	n.s.	In vivo	PRA	Arterial	5	Yes
Porter (1992)	Rat	Wistar‐Kyoto	n.s.	6–8 weeks	130–220 g	RPP: 117 ± 3	n.s.	In vivo	PRA	Arterial	5	No
	Rat	Wistar‐Kyoto	n.s.	6–8 weeks	130–220 g	RPP: 110 ± 2	n.s.	In vivo	PRA	Arterial	5	No
	Rat	Wistar‐Kyoto	n.s.	6–8 weeks	130–220 g	RPP: 114 ± 3	n.s.	In vivo	PRA	Arterial	5	No
	Rat	Wistar‐Kyoto	n.s.	6–8 weeks	130–220 g	RPP: 111.2	n.s.	In vivo	PRA	Arterial	7	No
	Rat	Sprague–Dawley	n.s.	6–8 weeks	180–240 g	RPP: 128 ± 5	n.s.	In vivo	PRA	Arterial	4	No
	Rat	Sprague–Dawley	n.s.	6–8 weeks	180–240 g	RPP: 119 ± 3	n.s.	In vivo	PRA	Arterial	4	No
	Rat	Sprague–Dawley	n.s.	6–8 weeks	180–240 g	n.s.	n.s.	In vivo	PRA	Arterial	6	No
	Rat	Sprague–Dawley	n.s.	6–8 weeks	180–240 g	n.s.	n.s.	In vivo	PRA	Arterial	9	No
Salomonsson et al. (1992)	Rabbit	New Zealand White	n.s.	n.s.	1500–2500 g	n.s.	Standard	Isolated	RR	n.s.	6	No
	Rabbit	New Zealand White	n.s.	n.s.	1500–2500 g	n.s.	Standard	Isolated	RR	n.s.	8	Yes
Scholz and Kurtz (1992)	Rat	SIV	Male	n.s.	250–350 g	n.s.	Standard	Isolated	RSR	Venous–Arterial difference	10	Yes
Scholz et al. (1993)	Rat	SIV	Male	n.s.	250–350 g	n.s.	Standard	Isolated	RSR	Venous–Arterial difference	15	No
Scholz et al. (1994)	Rat	Sprague–Dawley	Male	n.s.	250–350 g	n.s.	Standard	Isolated	RSR	Venous–Arterial difference	5	Yes
Scholz et al. (1994)	Rat	Sprague–Dawley	Male	n.s.	250–350 g	n.s.	Standard	Isolated	RSR	Venous–Arterial difference	5	Yes
Schweda et al. (2005)	Mouse	C57BL/6	Both	n.s.	23–30 g	n.s.	n.s.	Isolated	RSR	Venous	5	Yes
	Mouse	C57BL/6	Both	n.s.	23–30 g	n.s.	n.s.	Isolated	RSR	Venous	7	Yes
	Mouse	C57BL/6	Both	n.s.	23–30 g	n.s.	n.s.	Isolated	RSR	Venous	4	No
	Mouse	C57BL/6	Both	n.s.	23–30 g	n.s.	n.s.	Isolated	RSR	Venous	4	No
Seeliger et al. (1999)	Dog	Beagle	Female	2 years	12–19 kg	n.s.	5 mmol/kg/day	In vivo	PRA	Arterial	10	No
	Dog	Beagle	Female	2 years	12–19 kg	n.s.	Low salt diet	In vivo	PRA	Arterial	6	No
	Dog	Beagle	Female	2 years	12–19 kg	n.s.	5 mmol/kg/day	In vivo	PRA	Arterial	8	No
	Dog	Beagle	Female	2 years	12–19 kg	n.s.	5 mmol/kg/day	In vivo	PRA	Arterial	4	No
	Dog	Beagle	Female	2 years	12–19 kg	n.s.	5 mmol/kg/day	In vivo	PRA	Arterial	6	Yes
Villarreal et al. (1984)	Rat	Sprague–Dawley	Male	n.s.	250–350 g	RPP: 132 ± 4	Low salt diet	In vivo	PRA	Arterial	7	No
Wagner et al. (2007)	Mouse	Cx40/Wildtype	Both	12–20 weeks	n.s.	n.s.	n.s.	Isolated	RSR	Venous	5	Yes
Wagner et al. (2010)	Mouse	n.s.	n.s.	n.s.	n.s.	n.s.	n.s.	Isolated	RSR	Venous	4	No
Weaver et al. (1990)	Rat	Sprague–Dawley	Male	n.s.	352 ± 4 g	MAP: 99 ± 4	n.s.	In vivo	PRA	Arterial	8	No
Wende et al. (1993)	Rat	Wistar	Male	n.s.	250–300 g	n.s.	Standard	In vivo	PRA	Arterial	6	Yes
Wideman Jr et al. (1993)	Fowl	White leghorn	Male	30–32 weeks	2.18 ± 0.06 kg	RPP: 109 ± 5	n.s.	In vivo	PRA	Arterial	10	Yes

Abbreviations: BP, blood pressure; MA, meta‐analysis; MAP, mean arterial pressure; MRAP, mean renal arterial pressure; n.s, not specified; PRA, plasma renin activity; PRC, plasma renin concentration; RPP, renal perfusion pressure; RR, renin release; RSR, renin secretion rate.

Thirty studies with 36 comparisons could be included in the meta‐analysis for healthy animals. Of these comparisons, 33 were included in the main analysis and three additional comparisons were included in the subgroup analysis. Most comparisons used rats (*N* = 16) and dogs (*N* = 12). Experiments were either performed in vivo (*N* = 26) or in isolated kidneys (*N* = 10). Comparisons in dogs only used an in vivo experimental setup. Most comparisons used male animals (*N* = 17), some comparisons used mixed groups (*N* = 8) and only a few comparisons used female animals (*N* = 5). Comparisons reported their outcomes as PRA (*N* = 18), RR (*N* = 16), or PRC (*N* = 2). The baseline MAP of the healthy animals included in the meta‐analysis was 109 ± 11 mmHg.

#### 3.2.2 Hypertensive animals

Seven studies containing 13 comparisons using hypertensive animals were included (Table 2). The baseline MAP of these animals was higher as compared to the healthy comparisons (151 ± 10 vs. 109 ± 11 mmHg). Furthermore, a lower baseline renin value was observed in the hypertensive animals. All comparisons used rats, and most experiments were performed in vivo (*N* = 12). The animals were either male (*N* = 5) or the sex was not specified (*N* = 8). Renin was in most of the comparisons reported as PRA (*N* = 11); one comparison reported renin as PRC, and the other one as RR.

**TABLE 2:** Study characteristics of hypertensive comparisons.

Study	Species	Strain	Sex	Age	Weight	Baseline BP (mmHg)	Diet	Setup	Outcome	Sampling method	N	MA
Bertolino et al. (1996)	Rat	Lyon hypertensive	Male	14–16 weeks	n.s.	MRAP: 146	Standard	In vivo	PRC	Arterial	9	Yes
Braun et al. (1998)	Rat	Spontaneous hypertensive rat	Male	n.s.	300–400 g	MAP: 162 ± 6	Standard	In vivo	PRA	n.s.	6	Yes
Knoblich et al. (1996)	Rat	Sprague–Dawley (L‐NAME)	Male	n.s.	275–400 g	RPP: 156.6	Fasting	In vivo	PRA	Arterial	10	No
Medeiros et al. (1992)	Rat	Lyon hypertensive	Male	7 weeks	191 ± 8 g	n.s.	Standard	Isolated	RR	Venous	7	Yes
Porter (1990)	Rat	Spontaneous hypertensive rat	n.s.	6–9 weeks	120–175 g	RPP: 129.4	n.s.	In vivo	PRA	Arterial	5	No
	Rat	Spontaneous hypertensive rat	n.s.	6–9 weeks	120–175 g	RPP: 128.3	n.s.	In vivo	PRA	Arterial	8	No
	Rat	Spontaneous hypertensive rat	n.s.	6–9 weeks	120–175 g	RPP: 136.8	n.s.	In vivo	PRA	Arterial	11	No
	Rat	Spontaneous hypertensive rat	n.s.	14–16 weeks	210–320 g	RPP: 142 ± 3	n.s.	In vivo	PRA	Arterial	7	Yes
Porter (1992)	Rat	Spontaneous hypertensive rat	n.s.	6–8 weeks	120–200 g	RPP: 157 ± 3	n.s.	In vivo	PRA	Arterial	5	No
	Rat	Spontaneous hypertensive rat	n.s.	6–8 weeks	120–200 g	RPP: 160 ± 4	n.s.	In vivo	PRA	Arterial	5	No
	Rat	Spontaneous hypertensive rat	n.s.	6–8 weeks	120–200 g	RPP: 147.2 ± 8.0	n.s.	In vivo	PRA	Arterial	5	No
	Rat	Spontaneous hypertensive rat	n.s.	6–8 weeks	120–200 g	RPP: 154.2	n.s.	In vivo	PRA	Arterial	9	No
Wende et al. (1993)	Rat	Spontaneous hypertensive rat	Male	n.s.	250–300 g	n.s.	Standard	In vivo	PRA	Arterial	6	Yes

Abbreviations: BP, blood pressure; MA, meta‐analysis; MAP, mean arterial pressure; MRAP, mean renal arterial pressure; n.s, not specified; PRA, plasma renin activity; PRC, plasma renin concentration; RPP, renal perfusion pressure; RR, renin release; RSR, renin secretion rate.

Five studies with five comparisons could be included in the meta‐analysis for hypertensive animals. All comparisons used rats (*N* = 5): either spontaneous hypertensive rats (SHR), Lyon hypertensive (LH) rats, or L‐NAME treated Sprague–Dawley rats. Most of the animals were males (*N* = 4); one study did not specify the sex of the animals. The same study used an isolated experimental set up, whereas the other studies performed their experiments in vivo. Outcomes were reported as PRA (*N* = 3), PRC (*N* = 1), or PRC (*N* = 1).

### 3.3 Meta‐analysis

#### 3.3.1 Dose–response analysis in healthy animals

**FIGURE 3:**
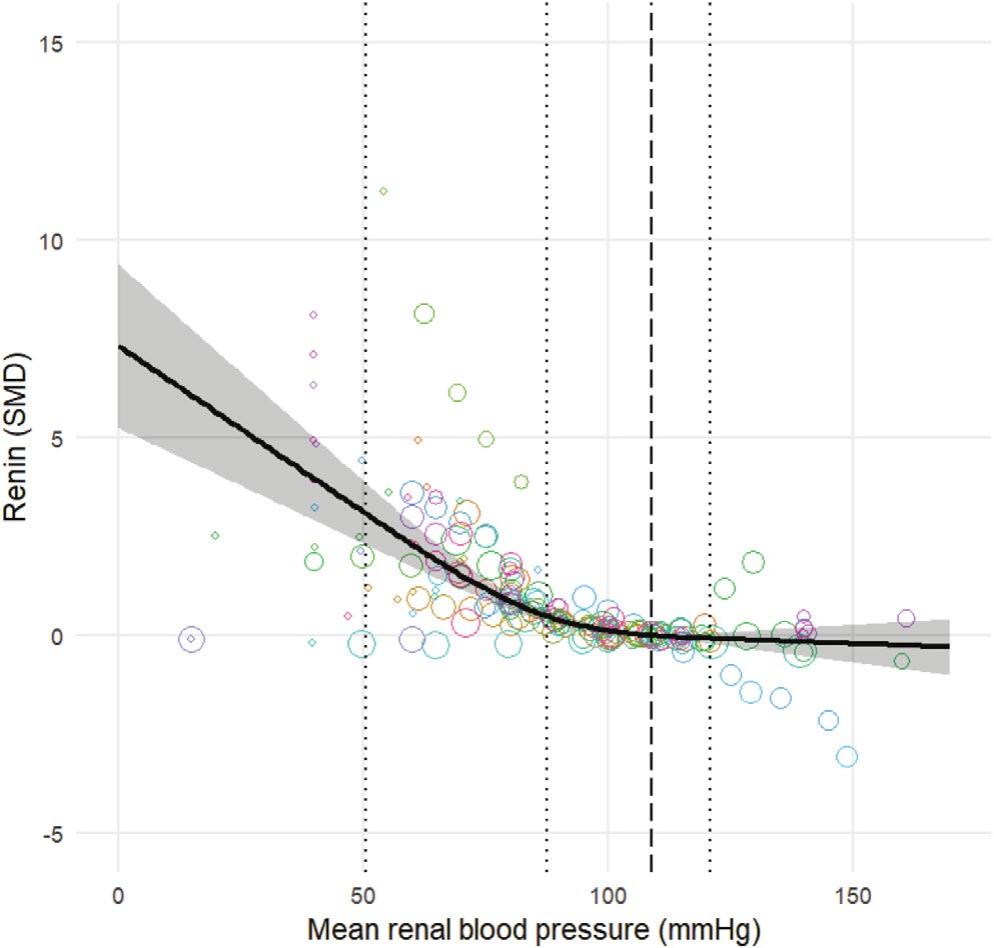
Dose–response relation between renal blood pressure and renin levels in healthy animals. Renin is expressed as the standardized mean difference (SMD) (black line) with the 95% confidence interval. The reference level is set to a renal blood pressure of 109 mmHg (black dashed line). Colored bubbles represent the estimated dose–response relation per individual comparison (*N* = 33). The dotted lines represent the knots for the restricted cubic spline model, located at 51 mmHg, 88 mmHg, and 121 mmHg.

**FIGURE 4:**
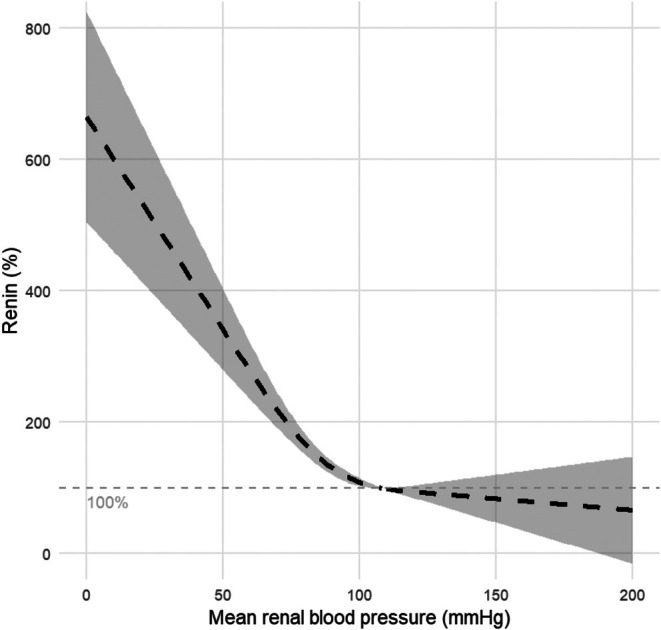
Dose–response relation between renal blood pressure and renin levels in healthy animals (*N* = 30). Renin is expressed as a percentage with a 95% confidence interval, where the renin values at the baseline MRAP of 109 mmHg are set to be 100%.

Figure 3 shows the result of the dose–response meta‐analysis in healthy animals; Figure 4 shows these results expressed as a percentage of the renin values at the baseline pressure. The mean baseline MRAP of the included animals was 109 mmHg. Renin decreases linearly up until an MRAP of 90–100 mmHg, whereafter a plateau level is reached. At this plateau, the renin level does not change anymore. The dose‐dependent relationship between renal pressure and renin release is comparable to what is observed in other studies included in the systematic review but were excluded from the meta‐analysis. Heterogeneity was observed (*I* ^2^ = 87.9%).

#### 3.3.2 Dose–response analysis in hypertensive animals

**FIGURE 5:**
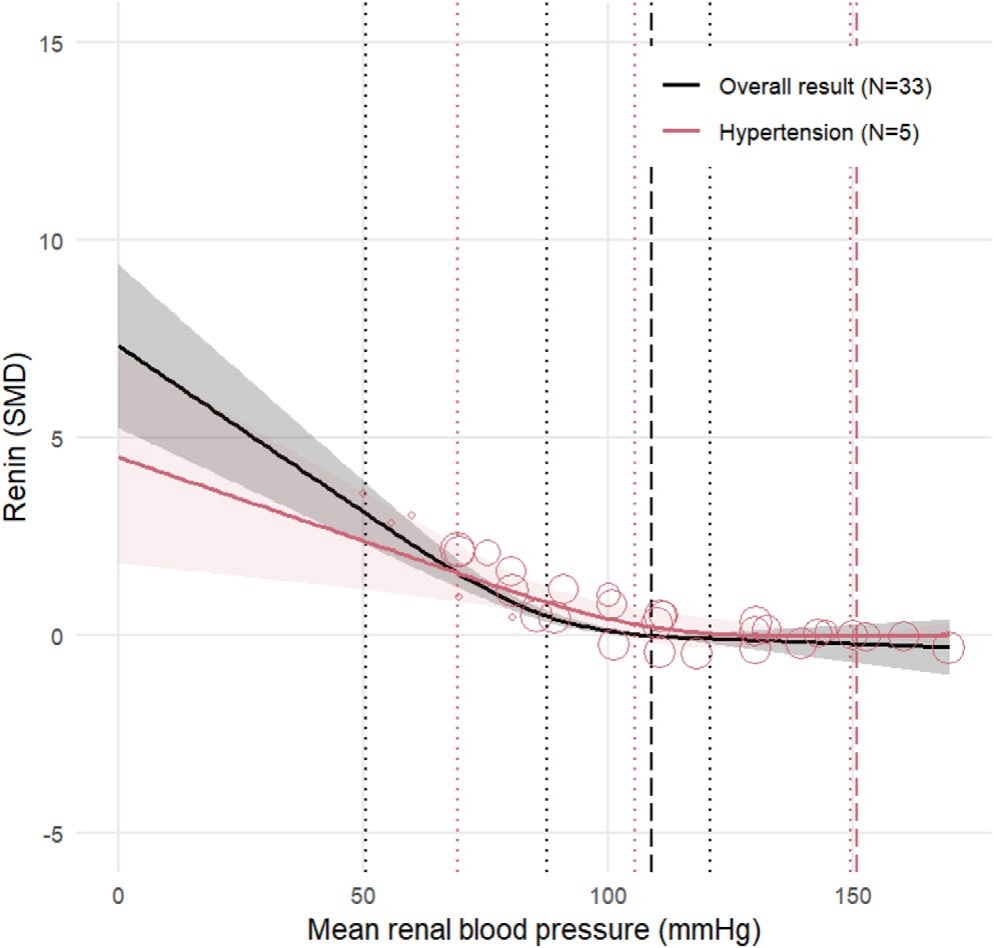
Dose–response relation between renal blood pressure and renin levels, determined for the overall result in healthy (black) and hypertensive (red) comparisons. Renin is expressed as the standardized mean difference (SMD) (solid lines) with the 95% confidence interval. The reference level is set to the mean baseline renal blood pressure per subgroup: 151 mmHg in hypertensive animals (red dashed line) and 109 mmHg for the overall result (black dashed line). The red bubbles represent the estimated dose–response relation of the included hypertensive comparisons. The dotted lines represent the knots for the restricted cubic spline model. The knots for the hypertensive data were 69, 106, and 150 mmHg.

**FIGURE 6:**
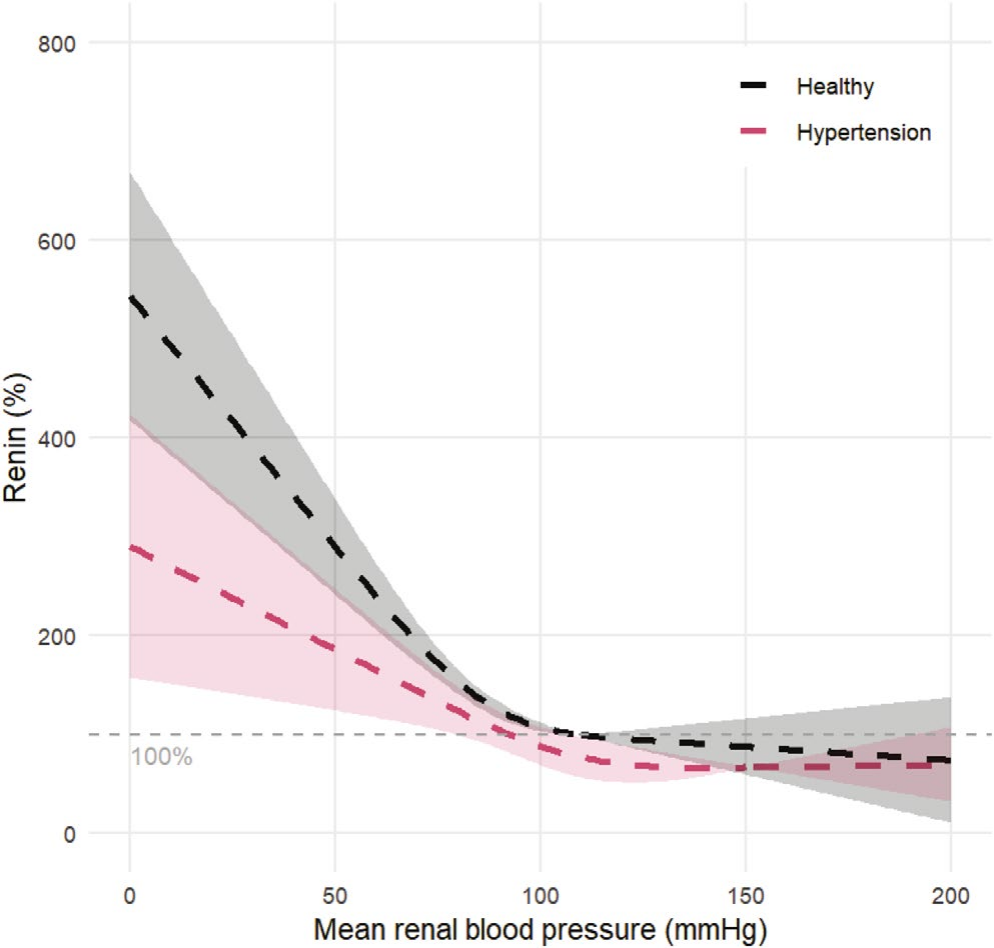
Dose–response relation between renal blood pressure and renin levels, determined for healthy (black) and hypertensive (red) comparisons. Renin is expressed in percentage (dashed lines) with the 95% confidence interval, where the renin values of healthy control animals at the baseline MRAP of 110 mmHg are set to be 100%. *N* = 5 for both groups.

Figure 5 shows the result of the dose–response meta‐analysis for hypertensive animals (*N* = 5) in comparison to healthy animals (*N* = 32). We observe that, similar to the response in healthy animals, renin linearly decreases as renal blood pressure increases. Between an MRAP of 105–115 mmHg, the linear decrease flattens whereafter a plateau level is reached. The linear slope appears to be less steep in hypertensive animals as compared to the healthy animals, and the threshold pressure seems to be higher. Since the SD and thus SMD can differ for hypertensive and healthy animals, a direct comparison between hypertensive and healthy animals can be seen in Figure 6. This figure contains data only from studies that compared hypertensive and healthy animals (*N* = 5). Renin is expressed as a percentage of the renin values at the baseline pressure (110 mmHg) in the healthy control animals. We still observe a difference in steepness of the linear decrease in renin with blood pressure. A decrease of 10 mmHg in renal blood pressure leads to an increase of 50 percentage points renin in healthy animals compared to an increase of 30 percentage points renin in hypertensive animals. The 95% confidence intervals of the healthy and hypertensive animals show minimal overlap in the linear region, indicating that this difference approaches statistical significance, although we were not able to test this statistically. Furthermore, we observe a visual difference in threshold pressure and plateau level.

#### 3.3.3 Meta‐analysis on threshold pressure and plateau levels

**FIGURE 7:**
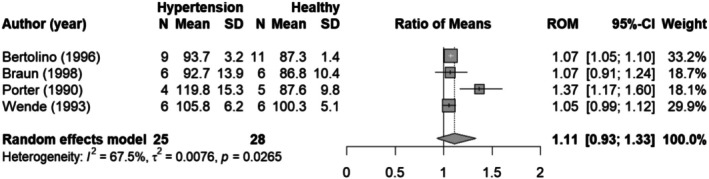
Forest plot of the ratio of means (ROM) of the threshold pressure in hypertensive animals compared to healthy animals. CI, confidence interval; *N*, number of animals; SD, standard deviation.

**FIGURE 8:**
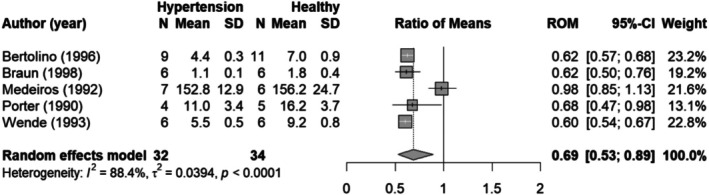
Forest plot of the ratio of means (ROM) of the renin plateau level in hypertensive animals compared to healthy animals. CI, confidence interval; *N*, number of animals; SD, standard deviation.

The threshold pressure determined based on the dose–response meta‐analysis (Figure 4) for the healthy animal group is 93 ± 2 mmHg. In hypertensive animals (Figure 6), the threshold pressure equals 106 ± 3 mmHg. To confirm the observed difference in threshold pressure and renin plateau level between healthy and hypertensive animals, we conducted an additional meta‐analysis for the individual studies. One study could not be included in the analysis on threshold pressure, as it measured limited data in the low blood pressure range (Medeiros et al., 1992). Figure 7 shows that the threshold pressure is not significantly different for hypertensive animals as compared to healthy animals (ROM 1.11; 95%‐CI [0.93–1.33]; *N* = 4; *I* ^2^ = 67%). Two comparisons showed a significant difference in threshold pressure for healthy compared to hypertensive animals, where the threshold pressure is higher in the hypertensive animals (Bertolino et al., 1996; Porter, 1990). The other two studies had the same direction of effect but did not reach significance. The renin plateau level was significantly lower in hypertensive animals as compared to healthy animals (ROM 0.69; 95% CI [0.53–0.89]; *N* = 5; *I* ^2^ = 88%) (Figure 8).

### 3.4 Risk of bias

#### 3.4.1 ROBINS‐I tool

**FIGURE E1:**
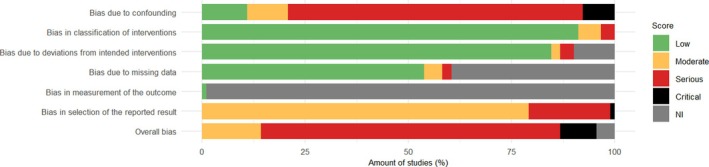
Risk of bias assessment by the ROBINS‐I tool for healthy comparisons included in the systematic review (*N* = 91).

**FIGURE E2:**
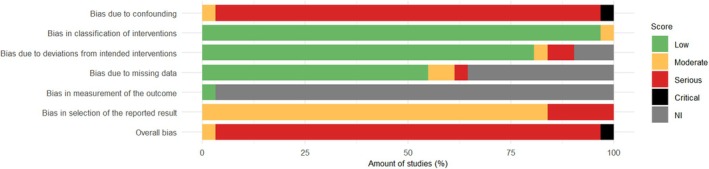
Risk of bias assessment by the ROBINS‐I tool limited to healthy comparisons included in the meta‐analysis (*N* = 33).

**FIGURE E3:**
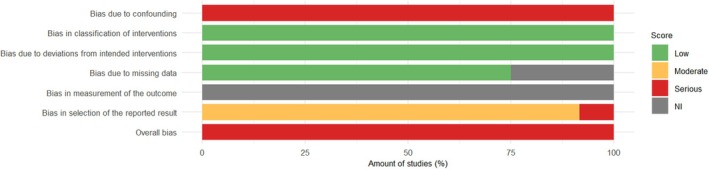
Risk of bias assessment by the ROBINS‐I tool for hypertensive comparisons included in the systematic review (*N* = 12).

**FIGURE E4:**
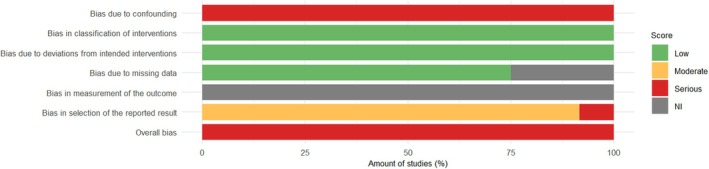
Risk of bias assessment by the ROBINS‐I tool limited to hypertensive comparisons included in the meta‐analysis (*N* = 5).

The risk of bias was assessed with the ROBINS‐I tool. Figure E1 (Appendix E) shows the results of the risk of bias assessment. For comparisons included in the systematic review, overall bias was scored to be at serious risk for most of the comparisons (*N* = 66, 73%). Most comparisons (*N* = 65, 71%) were scored to have a serious risk of bias due to confounding. There was low risk of bias in classification for interventions (*N* = 83, 91%) and bias due to deviations from intended interventions (*N* = 77, 85%). In almost all comparisons (99%), we were unable to score the bias in measurement of the outcome due to the lack of information. Comparisons included in the meta‐analysis (Appendix E, Figure E2) were more often scored to be at serious risk for bias due to confounding by the carryover effect (*N* = 29, 94%). The risk of bias in other domains was similar when we restricted the analysis to comparisons included in the meta‐analysis. The main difference is that comparisons that had a low risk of bias due to confounding could not be included in the meta‐analysis. This is mainly due to the fact that these comparisons did not have enough data points. In addition, we assessed risk of bias for the hypertensive comparisons (Appendix E, Figures E3 and E4), which shows similar results.

#### 3.4.2 SYRCLE RoB‐tool

**FIGURE E5:**
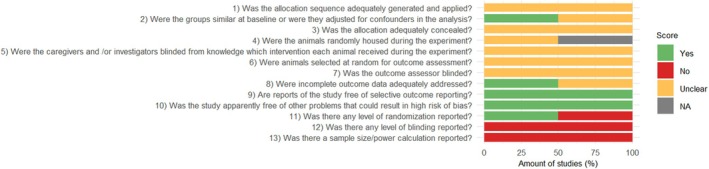
Risk of bias assessment by the SYRCLE tool for healthy comparisons included in the systematic review (*N* = 2).

**FIGURE E6:**
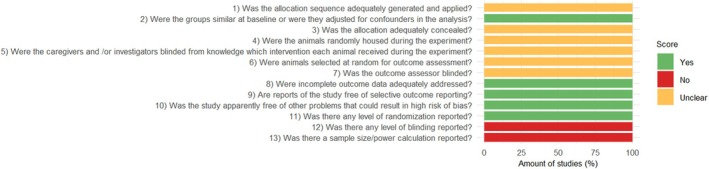
Risk of bias assessment by the SYRCLE tool limited to healthy comparison included in the meta‐analysis (*N* = 1).

**FIGURE E7:**
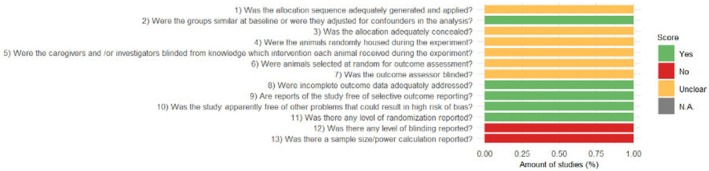
Risk of bias assessment by the SYRCLE tool limited to hypertensive comparison included in the meta‐analysis (*N* = 1).

Two control‐experimental comparisons (Knoblich et al., 1996; Nobiling et al., 1990) were assessed with the SYRCLE tool (Hooijmans et al., 2014) (Appendix E, Figure E5). For both comparisons, the majority of the risk of bias questions were assessed as unclear risk of bias. Furthermore, both studies did not report a power calculation or the level of blinding. One study did mention that the experimental animals were randomly subdivided into groups. This was also the study included in the meta‐analysis (Appendix E, Figure E6). The risk of bias assessment for the hypertensive comparison can be found in (Appendix E, Figure E7).

### 3.5 Subgroup and sensitivity analyses

**FIGURE F1:**
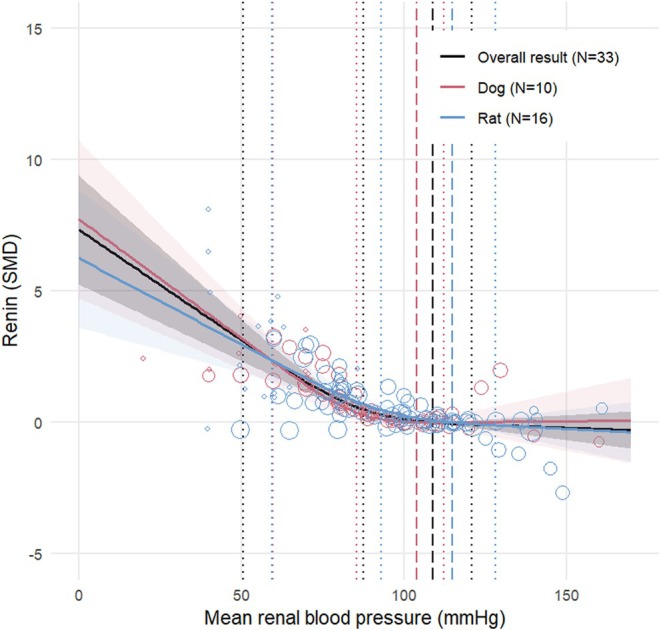
Dose–response relation between renal blood pressure and renin levels in healthy animals, determined for different species. Renin is expressed as the standardized mean difference (SMD) (solid lines) with the 95% confidence interval. The reference level is set to the mean baseline renal blood pressure per subgroup: 104 mmHg in dogs (red dashed line), 115 mmHg in rats (blue dashed line), and 109 mmHg for the overall result (black dashed line). Colored bubbles represent the estimated dose–response relation per individual comparison. The dotted lines represent the knots for the restricted cubic spline model. For the exact location of the knots, see Appendix D, Table D1.

**FIGURE F2:**
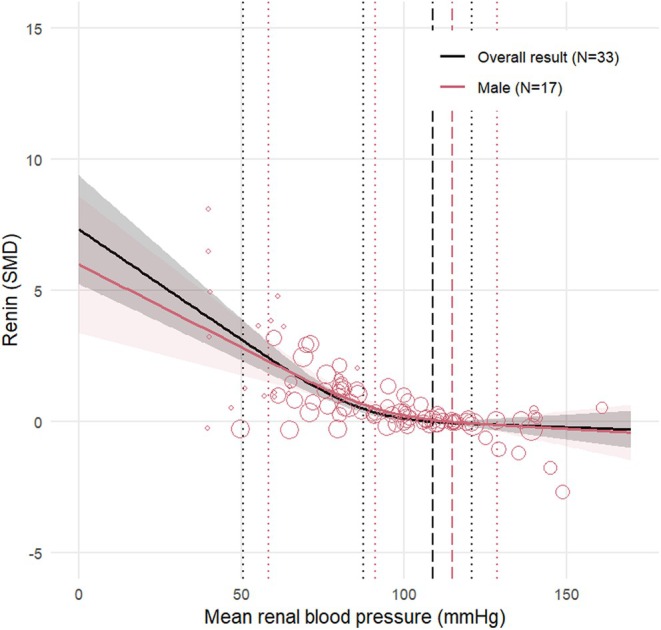
Dose–response relation between renal blood pressure and renin levels in healthy animals, determined for different sex. Renin is expressed as the standardized mean difference (SMD) (solid lines) with the 95% confidence interval. The reference level is set to the mean baseline renal blood pressure per subgroup: 115 mmHg for male animals (red dashed line) and 109 mmHg for the overall result (black dashed line). Colored bubbles represent the estimated dose–response relation per individual comparison. The dotted lines represent the knots for the restricted cubic spline model. For the exact location of the knots, see Appendix D, Table D1.

**FIGURE F3:**
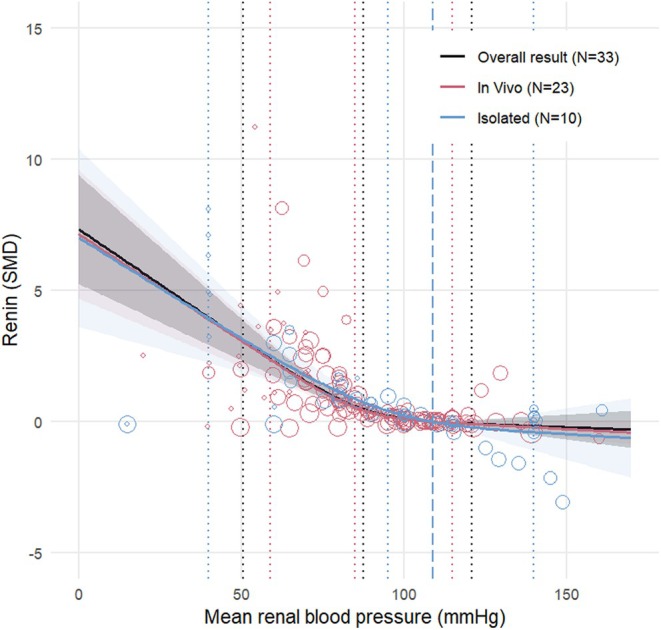
Dose–response relation between renal blood pressure and renin levels in healthy animals, determined for different experimental setup. Renin is expressed as the standardized mean difference (SMD) (solid lines) with the 95% confidence interval. The reference level is set to the mean baseline renal blood pressure per subgroup: 109 mmHg for the in vivo studies (red dashed line), 109 mmHg for the isolated studies (blue dashed line) and 109 mmHg for the overall result (black dashed line). Colored bubbles represent the estimated dose–response relation per individual comparison. The dotted lines represent the knots for the restricted cubic spline model. For the exact location of the knots, see Appendix D, Table D1.

**FIGURE F4:**
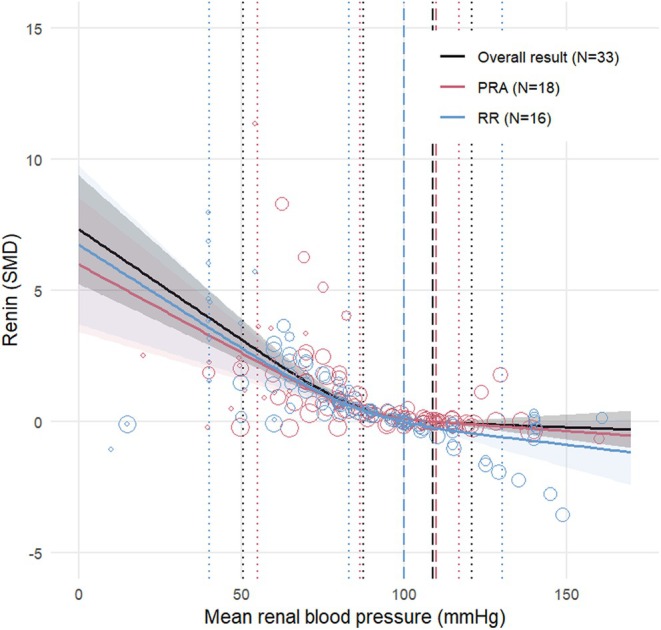
Dose–response relation between renal blood pressure and renin levels in healthy animals, determined for different reported outcomes. Renin is expressed as the standardized mean difference (SMD) (solid lines) with the 95% confidence interval. The reference level is set to the mean baseline renal blood pressure per subgroup: 112 mmHg for PRA (red dashed line), 100 mmHg for RR (blue dashed line), and 109 mmHg for the overall result (black dashed line). Colored bubbles represent the estimated dose–response relation per individual comparison. The dotted lines represent the knots for the restricted cubic spline model. For the exact location of the knots, see Appendix D, Table D1.

In case of 10 or more comparisons in a subgroup, subgroup analyses were conducted to explore heterogeneity caused by species, sex, experimental setup, and reported outcome (Appendix F, Figures F1, F2, F3, F4). The dose–response relation could only be assessed in the following subgroups: dogs, rats, males, in vivo and isolated experiments, renin reported as PRA and renin reported as RR, and did not seem to differ from the main result. Other subgroups contained too few studies for reliable subgroup analysis and were therefore not performed. An overview of the different reference values, knots, and heterogeneities per subgroup is displayed in Appendix D, Table D1.

**FIGURE F5:**
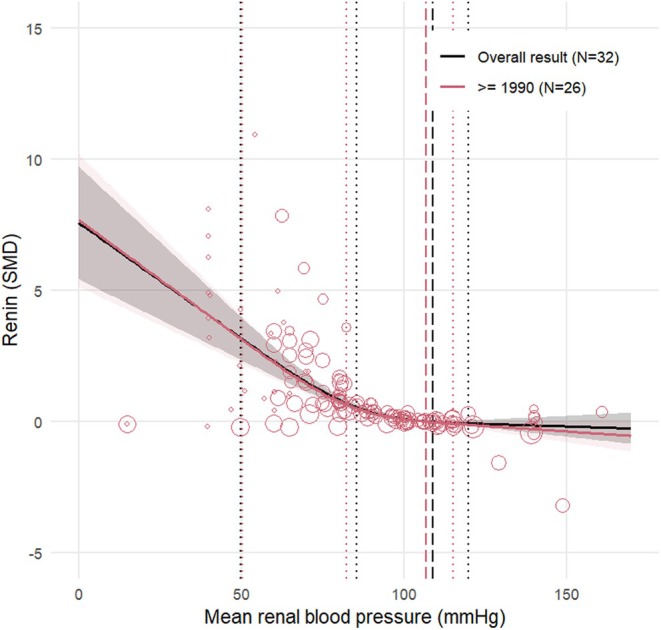
Dose–response relation between renal blood pressure and renin levels in healthy animals, determined for studies published after 1990. Renin is expressed as the standardized mean difference (SMD) (solid lines) with the 95% confidence interval. The reference level is set to the mean baseline renal blood pressure per subgroup: 108 mmHg in studies published after 1990 (red dashed line), and 109 mmHg for the overall result (black dashed line). Colored bubbles represent the estimated dose–response relation per individual comparison. The dotted lines represent the knots for the restricted cubic spline model. For the exact location of the knots, see Appendix D, Table D1.

**FIGURE F6:**
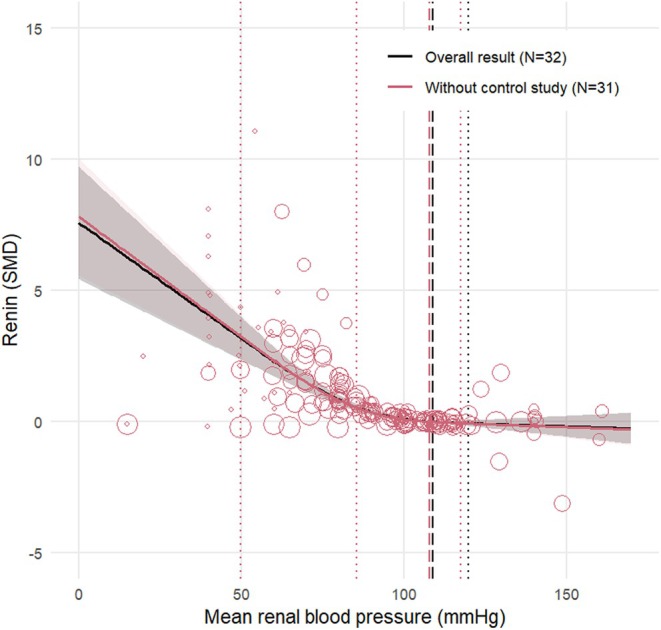
Dose–response relation between renal blood pressure and renin levels in healthy animals, and without the one control‐experiment study. Renin is expressed as the standardized mean difference (SMD) (solid lines) with the 95% confidence interval. The reference level is set to the mean baseline renal blood pressure per subgroup: 108 mmHg for comparisons without the one control‐experiment study (red dashed line), and 109 mmHg for the overall result (black dashed line). Colored bubbles represent the estimated dose–response relation per individual comparison. The dotted lines represent the knots for the restricted cubic spline model. For the exact location of the knots, see Appendix D, Table D1.

**FIGURE F7:**
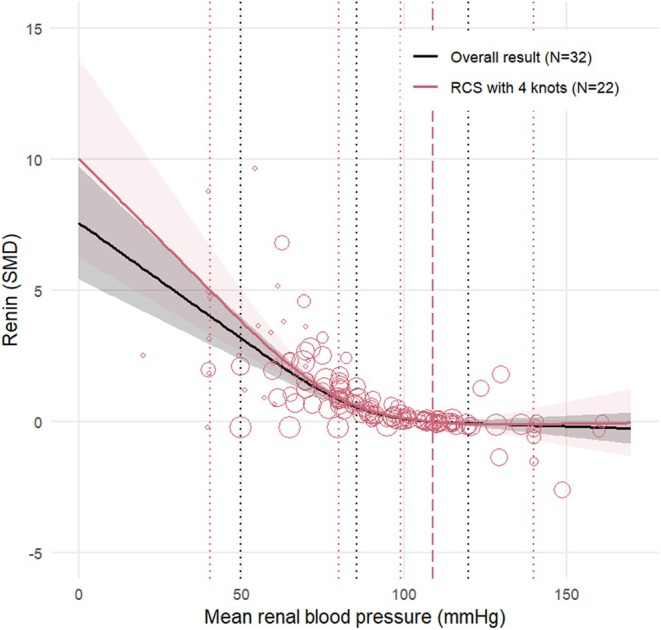
Dose–response relation between renal blood pressure and renin levels in healthy animals with a 3‐knots RCS‐model (black) and a 4‐knots RCS‐model (red). Renin is expressed as the standardized mean difference (SMD) (solid lines) with the 95% confidence interval. The reference level is set to the mean baseline renal blood pressure per subgroup: 109 mmHg for both the 3‐knots model and 4‐knots model. Colored bubbles represent the estimated dose–response relation per individual comparison. The dotted lines represent the knots for the restricted cubic spline model. For the exact location of the knots, see Appendix D, Table D1.

We performed sensitivity analyses to evaluate the influence of publication year (Appendix F, Figure F5), leaving out the one comparison with an independent control group (Appendix F, Figure F6), and type of RCS‐model (Appendix F, Figure F7) on the outcome of the dose–response meta‐analysis. The predicted dose–response relation between renal blood pressure and renin was not influenced by restricting the included studies to studies published after the year 1990 or leaving out the one comparison with an independent control. Furthermore, we compared the RCS‐model with 3 knots to an RCS‐model with 4 knots. The 3‐knots model had the best fit, as both the AIC and BIC were lower (Appendix F, Table F1). There is large overlap in the 95% confidence intervals of the 3‐knots and 4‐knots model, indicating no significant difference. Important to note is that only 23 comparisons were included in the 4‐knots RCS‐model, as a minimum of 5 datapoints per comparison was required to perform this analysis. As almost all studies were scored to have a serious risk of bias, we were not able to distinguish different subgroups to evaluate in the sensitivity analysis.

## 4 DISCUSSION

The renal baroreflex is one of the activators of the volume‐regulatory renin‐angiotensin‐aldosterone system. To assess the dose‐dependent relation between renal pressure and renin release, we performed a systematic literature review and dose–response meta‐analysis of animal studies. In this analysis, we observed a linear decrease in renin as renal blood pressure increases until a threshold pressure is reached, in both hypertensive and healthy animals. Above this blood pressure level, renin reaches a plateau level. Hypertensive animals show impaired renin release to renal blood pressure drop, a statistically comparable threshold but a lower plateau.

In healthy animals, we observed a linear decrease in renin until the threshold pressure of 93 mmHg, whereafter a plateau level is reached. This threshold pressure is similar to what was observed in earlier literature (Bertolino et al., 1994; Ehmke et al., 1992; Farhi et al., 1982, 1987; Imagawa et al., 1984; Kirchheim et al., 1987; Porter, 1990, 1992; Wende et al., 1993; Wideman Jr et al., 1993). Furthermore, we performed several subgroup analyses to explore heterogeneity. None of the conducted subgroup analyses revealed a different relation between renin levels and blood pressure, strengthening our conclusion that renin release decreases linearly as renal blood pressure increases until it reaches the threshold pressure, after which a plateau level is reached. Studies included in the systematic review but not in the meta‐analysis also observe a decrease in renin when renal blood pressure rises. Often, these studies did not have enough datapoints to observe the plateau and threshold as well.

In hypertensive animals, we also observed a linear decrease in renin until a threshold pressure, whereafter renin levels reach a plateau. The linear decline appears to be less steep in hypertensive animals compared to healthy animals. This difference was presumably not significant (slight overlap in 95% CI), probably due to the small number of comparisons (*N* = 5). The study of Knoblich et al. could not be included in the meta‐analysis but also describes a diminished renal baroreflex in hypertensive animals as compared to normotensive animals (Knoblich et al., 1996). Furthermore, our dose–response meta‐analysis showed that the threshold pressure appears to be higher in hypertensive animals (106 mmHg) as compared to healthy animals (93 mmHg). This is in agreement with the studies of Ehmke et al., who observed elevations in mean arterial pressure with an increased threshold pressure of the RSRC (Ehmke et al., 1987a, 1987b). Nevertheless, our post hoc meta‐analysis regarding threshold pressure did not show a significant difference, which might be the result of the low number of studies and very few animals included per study. We did observe a significant difference in the height of plateau renin levels, which is in agreement with earlier research, as former studies also describe lower basal renin values in hypertensive animals as compared to healthy animals (Bertolino et al., 1996; Braun et al., 1998; Knoblich et al., 1996; Medeiros et al., 1992; Porter, 1990, 1992; Wende et al., 1993). It has to be kept in mind that we were only able to include five comparisons in our meta‐analysis on hypertensive animals, so more data is necessary to draw a robust conclusion.

Several pathways involving the juxtaglomerular or macula densa cells may contribute to the altered renin response to blood pressure in hypertensive animals included in our meta‐analysis (SHR, LH, and L‐NAME treated rats). Hypertension relates to preglomerular vasoconstriction (Braun et al., 1998; Knoblich et al., 1996; Kost Jr. & Jackson, 1993), hypertrophy of the afferent arterioles (Baldwin & Neugarten, 1987; Mennuni et al., 2014), enhanced vasoconstrictive response to angiotensin II (Harrison‐Bernard et al., 1997; Kost Jr. & Jackson, 1993; Kovács et al., 2002; Rivera‐Jardón et al., 2009; Schweda & Kurtz, 2011), impaired natriuresis (Farhi et al., 1983; Johnson et al., 2005; Knoblich et al., 1996; Liu et al., 1996; Medeiros et al., 1992; Takenaka et al., 1990), and glomerular injuries and persistent renal tissue damage (Baldwin & Neugarten, 1987; Johnson et al., 2005; Marín et al., 2005; Mennuni et al., 2014). Each of the described mechanisms might contribute to a greater or lesser extent to the plateau, threshold, and pressure to renin release relation as observed in hypertensive animals.

## 5 STRENGTHS AND LIMITATIONS

To our knowledge, this paper presents the first systematic review and meta‐analysis on the renal baroreflex in healthy and hypertensive animals. We used a systematic approach with clearly defined inclusion and exclusion criteria for study selection, and therefore we believe that we were able to include and evaluate all peer‐reviewed manuscripts regarding this topic. Furthermore, we performed an extensive dose–response meta‐analysis with multiple subgroup and sensitivity analyses to assess the potential effects of various study design‐related characteristics on renin release.

Nevertheless, we want to discuss some important limitations of this study. First, we were not able to perform the meta‐analysis on absolute renin values, because renin was reported as either PRC, PRA, and RR, and the magnitude of each outcome depends on the renin assay used. However, we were able to convert the relative dose–response relation between renal blood pressure and renin release to percental values, which improved the clinical interpretability.

Second, the studies that were included in this review used mostly male animals or groups with both sexes. Only 10 comparisons (11%) used females, of which we were only able to include five in our meta‐analysis. Therefore, we were not able to perform a subgroup analysis regarding the effect of female sex. The underrepresentation of female animals in this research field reduces the external validity. On the other hand, we included multiple animal species and strains in the meta‐analysis, and we did observe similar dose–response in our subgroup analyses, enhancing the reliability of evidence of this effect in humans. We recommend to conduct more studies in female animals to further explore the effect of sex on renal baroreflex mechanisms.

Third, in our risk of bias assessment, we observed a moderate to critical risk of bias due to confounding in most of the included studies. This is due to the fact that most studies decreased blood pressure subsequently, without returning to a baseline pressure in between steps. This way, the effect of a previous blood pressure step could still affect the following blood pressure step. However, it has been shown that renin reaches its peak already after 5 min of renal blood pressure alteration (Medeiros et al., 1992; Nafz et al., 1997). As all studies measured renin more than 5 min after the change in blood pressure, this suggests there is not too much influence of a carryover effect. Furthermore, many studies inadequately reported essential methodological details, resulting in an unclear risk of bias in most risk of bias domains. This hampers drawing reliable conclusions from the included animal studies. Animal studies should improve their reporting quality of essential methodological details and adhere to the ARRIVE guidelines (Percie du Sert et al., 2020) which will improve the reliability of systematic reviews, but also the reproducibility and validity of the conducted research.

Fourth, we observed substantial heterogeneity between the included studies. Although we tried to explain the observed heterogeneity with subgroup analyses, this was not always possible due to the low number of studies included in the review. Nevertheless, the subgroup analyses that could be conducted did not reveal significant differences between subgroups. It is important, however, to be aware that, due to the explorative nature of experimental animal studies, heterogeneity is expected and sometimes even deliberately introduced (Hooijmans et al., 2018).

## 6 CONCLUSION

In summary, we observed that the healthy renal baroreflex is characterized by a linear decrease in renin levels as renal blood pressure rises, until the threshold blood pressure is reached. Above this blood pressure threshold, renin output stabilizes at a plateau level. In hypertensive animals, the linear slope is diminished, and the plateau level seems to be slightly decreased as compared to healthy animals. The results of our study provide valuable insight in the dose‐dependent relation between renal pressure and renin release, and its adjustments during hypertension. These findings can be used to improve our ability to predict and anticipate on conditions that affect blood pressure and renin release.

## FUNDING INFORMATION

This study was funded by the ZonMw “Meer Kennis met Minder Dieren” program (grant number: 01142042330009).

## CONFLICT OF INTEREST STATEMENT

The authors declare no competing interests.

## Data Availability

The data that support the findings of this study are available from the corresponding author upon reasonable request.

## APPENDIX A Search strategy

**Table:**

Component	PubMed search term	Embase search term
Kidney / renal circulation	kidney[MeSH] OR “renal circulation”[MeSH] OR “renal artery”[MeSH] OR kidney*[Title/Abstract] OR renal*[Title/Abstract]	exp kidney/ OR kidney circulation/ OR (kidney* OR renal*).ti,ab,kf.
Renin	“renin”[MeSH] OR “renin”[Title/Abstract] OR “angiotensin forming enzyme”[Title/Abstract] OR “angiotensinogenase”[Title/Abstract] OR renine[Title/Abstract] OR prorenin[Title/Abstract] OR prerenin[Title/Abstract] OR preprorenin[Title/Abstract] OR “pre‐prorenin” [Title/Abstract] OR “pre prorenin” [Title/Abstract] OR cryorenin[Title/Abstract]	renin/ OR plasma renin activity/ OR kidney renin/ OR tetradecapeptide renin/ OR renin release/ OR prorenin/ OR (renin OR angiotensin forming enzyme OR angiotensinogenase OR renine OR prorenin OR prerenin OR preprorenin OR pre‐prorenin OR cryorenin).ti,ab,kf.
Blood pressure	“blood pressure”[MeSH] OR pressure[Title/Abstract]	exp blood pressure/ OR pressure.ti,ab,kf.
Baroreceptor	baroreflex[MeSH] OR pressoreceptor[MeSH] OR baroreceptor*[Title/Abstract] OR baroreflex[Title/Abstract] OR baroreflexes[Title/Abstract] OR “pressure dependent”[Title/Abstract] OR “stimulus response”[Title/Abstract] OR “stimulus responses”[Title/Abstract] OR pressoreceptor*[Title/Abstract] OR “pressor reflex*”[Title/Abstract] OR “pressure reflex*”[Title/Abstract] OR “pressor response”[Title/Abstract] OR “pressure response”[Title/Abstract] OR “pressor responses”[Title/Abstract] OR “pressure responses”[Title/Abstract] OR “stretch receptor*”[Title/Abstract]	baroreceptor/ OR pressoreceptor reflex/ OR stretch receptor/ OR pressor response/ OR (baroreceptor* OR baroreflex OR baroreflexes OR pressure dependent OR stimulus response OR stimulus responses OR pressoreceptor* OR pressor reflex* OR pressure reflex* OR pressor response OR pressor responses OR pressure response OR pressure responses OR stretch receptor*).ti,ab,kf.

## APPENDIX B Tools for risk of bias assessment

### B.1 ROBINS‐I tool

**Table:**

Signaling questions	Possible answers	Remarks
Bias due to confounding		
1.1 Is there potential for confounding of the effect of intervention in this study?	Y/PY/ PN/N	
Ø If N/PN to 1.1: the study can be considered to be at low risk of bias due to confounding and no further signaling questions need be considered		
Ø If Y/PY to 1.1: determine whether there is a need to assess time‐varying confounding:		
1.2 Were the measurements of outcome made at sufficient pre‐intervention time points to permit characterization of pre‐intervention trend and patterns?	Y/PY/ PN/N /NI	
1.3 Were there extraneous events or changes in context around the time of intervention that could have influenced the outcome?	Y/PY/ PN/N /NI	
1.4 Did the study authors use an appropriate analysis method that accounts for time trend and patterns, and controls for all the important confounding domains?	Y/PY/ PN/N/NI	
What is the predicted direction of bias due to confounding?	Low/Moderate/Serious/Critical/NI	
Bias in classification of interventions		
3.1 Were intervention groups clearly defined?	Y/PY/ PN/N/NI	
3.2 Was the information used to define intervention groups recorded at the start of the intervention?	Y/PY/ PN/N/NI	
What is the predicted direction of bias due to measurement of outcomes or interventions?	Low/Moderate/Serious/Critical/NI	
Bias due to deviations from intended interventions		
4.1. Was there appropriately accounted for the effects of any preparatory (pre‐interruption) phases of the intervention?	Y/PY/ PN/N/NI	
What is the predicted direction of bias due to deviations from the intended interventions?	Low/Moderate/Serious/Critical/NI	
Bias due to missing data		
5.1 Were outcome data available for all, or nearly all, animals?	Y/PY/ PN/N/NI	
5.2 Were animals excluded due to missing data on intervention status?	Y/PY/ PN/N/NI	
5.3 Were animals excluded due to missing data on other variables needed for the analysis?	Y/PY/ PN/N/NI	
5.4 If PN/N to 5.1, or Y/PY to 5.2 or 5.3: Are the proportion of animals and reasons for missing data similar across interventions?	NA/Y/PY/ PN/N/NI	
5.5 If PN/N to 5.1, or Y/PY to 5.2 or 5.3: Is there evidence that results were robust to the presence of missing data?	NA/Y/PY/ PN/N/NI	
5.6 Was outcome data missing for whole groups of animals as well as individual animals?	Y/PY/ PN/N/NI	
What is the predicted direction of bias due to missing data?	Low/Moderate/Serious/Critical/NI	
Bias in measurement of the outcome		
6.1 Could the outcome measure have been influenced by knowledge of the intervention received?	Y/PY/ PN/N/NI	
6.2 Were outcome assessors aware of the intervention received by study animals?	Y/PY/ PN/N/NI	
6.3 Were the methods of outcome assessment comparable across intervention groups?	Y/PY/ PN/N/NI	
6.4 Were the methods of outcome assessment comparable before and after the intervention?	Y/PY/ PN/N/NI	
6.5 Were any systematic errors in measurement of the outcome related to intervention received?	Y/PY/ PN/N/NI	
What is the predicted direction of bias due to measurement of outcomes?	Low/Moderate/Serious/Critical/NI	
Bias in selection of the reported result		
*Is the reported effect estimate likely to be selected, on the basis of the results, from…*		
7.1 … multiple outcome measurements within the outcome domain?	Y/PY/ PN/N/NI	
7.2 … multiple analyses of the intervention‐outcome relationship?	Y/PY/ PN/N/NI	
7.3 … different subgroups?	Y/PY/ PN/N/NI	
What is the predicted direction of bias due to selection of the reported result?	Low/Moderate/Serious/Critical/NI	

Abbreviations: Y, yes; PY, probably yes; N, no; PN; probably no; NI, no information. Colours in answer possibilities correspond with low (green) or high (red) risk of bias.

### B.2 SYRCLE tool

Reporting questions:

**Table:**

#	Signaling question	Answer question	Risk of bias	Motivation / comments
1	Was the allocation sequence adequately generated and applied?	Yes/No/Unclear	Low/High/Unclear	
2	Were the groups similar at baseline or were they adjusted for confounders in the analysis?	Yes/No/Unclear	Low/High/Unclear	
3	Was the allocation adequately concealed?	Yes/No/Unclear	Low/High/Unclear	
4	Were the animals randomly housed during the experiment?	Yes/No/Unclear	Low/High/Unclear	
5	Were the caregivers and /or investigators blinded from knowledge which intervention each animal received during the experiment?	Yes/No/Unclear	Low/High/Unclear	
6	Were animals selected at random for outcome assessment?	Yes/No/Unclear	Low/High/Unclear	
7	Was the outcome assessor blinded?	Yes/No/Unclear	Low/High/Unclear	
8	Were incomplete outcome data adequately addressed?	Yes/No/Unclear	Low/High/Unclear	
9	Are reports of the study free of selective outcome reporting?	Yes/No/Unclear	Low/High/Unclear	
10	Was the study apparently free of other problems that could result in high risk of bias?	Yes/No/Unclear	Low/High/Unclear	

Colours in answer possibilities correspond with low (green), high (red) or unclear (yellow) risk of bias.

1. Was there any level of randomization reported? Yes/No
2. Was there any level of blinding reported? Yes/No
3. Was there a sample size and power calculation reported?Yes/No

## APPENDIX C Study characteristics

Overview of the study characteristics of comparisons included in the systematic review (SR, left) and limited to comparisons included in the meta‐analysis (MA, right).

## APPENDIX D Model properties

**TABLE D1:** Model properties to determine and plot dose–response relation between renal blood pressure and renin in healthy and hypertensive animals.

Analysis	*N*	BP_ref_ (mmHg)	Knots (mmHg)	*I* ^2^ (%)
Main	33	109	51; 88; 121	87.9
Hypertensive	5	151	70; 106; 150	62.4
Rats	16	115	59; 93; 128	88.6
Dogs	10	104	60; 85; 112	70.4
Males	17	115	59; 91; 129	88.6
In vivo	23	109	59; 85; 115	84.2
PRA	18	110	55; 87; 117	81.7
RR	16	100	40; 83; 130	89.1
Publication year	26	107	50; 82; 115	85.2
Study type	32	108	50; 88; 120	88.2
RCS with 4 knots	23	109	44; 80; 100; 140	86.6

Abbreviations: BP_ref_, reference blood pressure; *N*, number of animals; PRA, plasma renin activity; RCS, restricted cubic splines; RR, renin release.

## APPENDIX F Subgroup and sensitivity analyses

### F.7 Sensitivity analysis: RCS‐model with four knots

**TABLE F1:** 4 knots versus 3 knots.

Model	AIC	BIC
RCS with 3 knots	−212	−201
RCS with 4 knots	−59	−39

*Note*: AIC and BIC scores for the evaluated statistical models in the sensitivity analysis.

Abbreviations: AIC, Akaike Information Criterion; BIC, Bayesian Information Criterion; RCS, restricted cubic splines.
